# Advances in Recellularization of Decellularized Liver Grafts with Different Liver (Stem) Cells: Towards Clinical Applications

**DOI:** 10.3390/cells12020301

**Published:** 2023-01-13

**Authors:** Burak Toprakhisar, Catherine M. Verfaillie, Manoj Kumar

**Affiliations:** Stem Cell Institute, Department of Stem Cell and Developmental Biology, KU Leuven, 3000 Leuven, Belgium

**Keywords:** regenerative medicine, liver tissue engineering, organ engineering, liver niche, stem cells, hepatocytes, endothelial cells

## Abstract

Liver transplantation is currently the only curative therapy for patients with acute or chronic liver failure. However, a dramatic gap between the number of available liver grafts and the number of patients on the transplantation waiting list emphasizes the need for valid liver substitutes. Whole-organ engineering is an emerging field of tissue engineering and regenerative medicine. It aims to generate transplantable and functional organs to support patients on transplantation waiting lists until a graft becomes available. It comprises two base technologies developed in the last decade; (1) organ decellularization to generate a three-dimensional (3D) extracellular matrix scaffold of an organ, and (2) scaffold recellularization to repopulate both the parenchymal and vascular compartments of a decellularized organ. In this review article, recent advancements in both technologies, in relation to liver whole-organ engineering, are presented. We address the potential sources of hepatocytes and non-parenchymal liver cells for repopulation studies, and the role of stem-cell-derived liver progeny is discussed. In addition, different cell seeding strategies, possible graft modifications, and methods used to evaluate the functionality of recellularized liver grafts are outlined. Based on the knowledge gathered from recent transplantation studies, future directions are summarized.

## 1. Introduction

The liver is the largest internal organ in the human body. Apart from vital functions such as protein synthesis, blood detoxification, and drug biotransformation, it has a remarkable regenerative capacity. A delicate interplay between hepatocytes, non-parenchymal liver cells, and the surrounding extracellular matrix (ECM) maintains these metabolic functions under normal homeostatic conditions [1]. However, external hepatic insults such as viral infections, toxic drugs, long-term alcohol abuse, and certain metabolic and genetic disorders can disrupt liver homeostasis. When such persistent hepatic insults outpace the regenerative capacity of liver, it leads to the initiation of progressive liver degeneration.

Progressive liver degeneration occurs as a result of chronic damage or acute damage (e.g., acetaminophen overload), which may result in liver failure. Subsequent loss of function, termed End-Stage Liver Disease (ESLD), causes two million deaths per year worldwide [2]. Combined, they account for 3.5% of all deaths. Additionally, as a result of the progressively increasing worldwide obesity epidemic, which causes non-alcoholic steatohepatitis (NASH) and associated NASH, the liver fatality rate is expected to increase continuously in the coming years.

In addition to high mortality, ESLDs cause a variety of extrahepatic morbidities, reduce quality of life, and burden the healthcare system worldwide [2]. Although several candidate drugs have been developed to reverse and/or prevent the progression of liver degeneration (e.g., fibrotic and inflammatory state), there is no approved medication to counteract these processes as yet [3]. This lack of effective medication leaves whole organ transplantation as the only curative therapy for ESLD patients, which has propelled advancements in the field of regenerative medicine and tissue engineering to generate alternative solutions.

**Figure 1:**
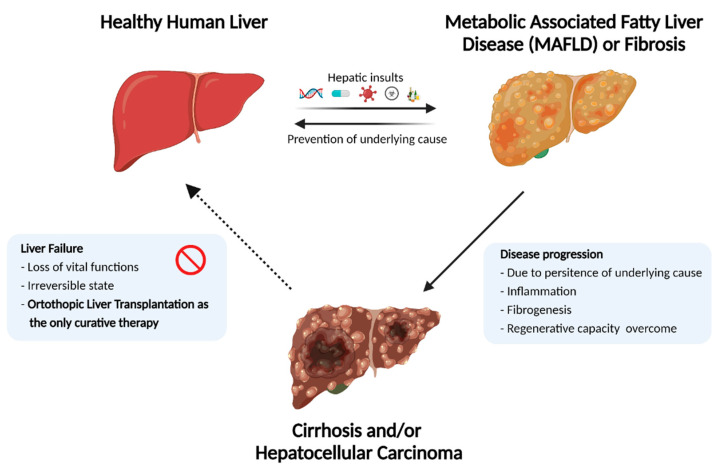
Progression to End-Stage Liver Disease. Inborn metabolic disorders or hepatic insults such as drug chronic viral infection, exposure to toxic chemicals, alcohol abuse, and fatty diet may result in a fibrotic and/or inflammatory liver. Persistence of hepatic insults may overcome the inherent regenerative capacity of the liver, resulting in liver failure. As this state is irreversible, the only curative therapy is orthotopic liver transplantation.

**Figure 2:**
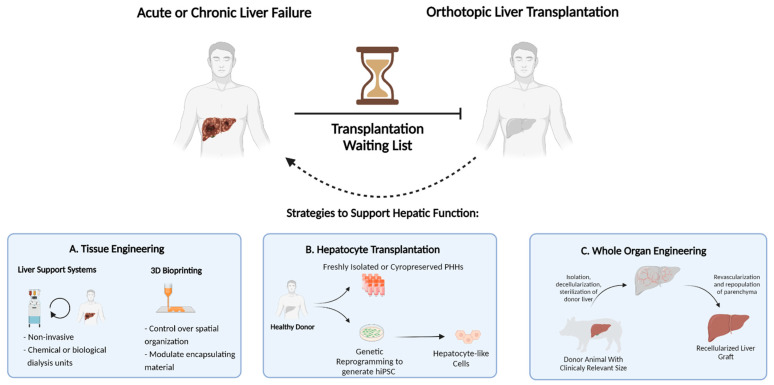
Solutions to expand the lifetime of patients on transplantation waiting list. Hepatocyte transplantation, liver support systems and liver-mimicking artificial constructs, as well as whole-organ engineering, have been proposed to expand the lifetime of the patients with acute or chronic liver failure until suitable graft becomes available.

Orthotopic liver transplantation, wherein the diseased liver is replaced with a healthy donor liver, is the only curative therapy for patients with ESLD. (Figure 1) Unfortunately, this option is limited to only a small fraction of patients due to the scarcity of available donor organs and immunological incompatibility upon transplantation. According to the 2018 annual liver transplantation statistics of the Scientific Registry of Transplant Recipients (SRTR, US), approximately 12,000 patients were registered on the waiting list, while only 8250 liver transplantations were performed. Thus, despite the increased success of transplantations performed in recent years, more than 4000 registered patients did not receive a donor liver [4]. This dramatic gap between the number of available donor organs and patients eligible for transplantation necessitates therapeutic alternatives. Over the last few decades, remarkable developments in the field of regenerative medicine and tissue engineering have proposed strategies to increase the lifetime of ESLD patients [5]. This includes strategies such as external liver support systems, cell transplantation, three-dimensional mini-tissue implantations, and, most notably, whole-organ engineering. (Figure 2)

## 2. Tissue Engineering Solutions

### 2.1. Artificial Liver Platforms

Artificial liver support (ALS) devices are the earliest example of tissue engineering concepts used to bridge patients requiring liver grafts until a donor liver becomes available or the liver recovers from the insult. They achieve this by providing essential liver function externally. ALS devices were developed in the early 1990s and essentially aim to remove toxins from the patient plasma via external adsorption and filtration systems. Subsequent iterations of ALS devices also made it possible to take over other essential liver functions, such as removal of bile acids, phenols, glycoside derivatives, and excess fatty acids [6]. In particular, the Molecular Adsorbent Recirculation System (MARS) has been proposed as an effective method to improve both biochemical parameters and hepatic encephalopathy in patients. A meta-data analysis demonstrated that compared to standard medical treatment, MARS treatments not only significantly enhanced patient survival (up to 74%, n = 165), but also reduced the severity of hepatic encephalopathy [7]. 

A step forward from ALS devices are Bioartificial Livers (BALs), wherein the chemical filtration units were replaced by hepatocytes either from xenogeneic or human origin, to detoxify patients’ blood. The main advantage of BALs over ALS devices is that BALs can detoxify the blood from patients more effectively, and most notably, it can synthesize liver-specific proteins and hepatotropic factors [8]. A recent study by Chen et al. demonstrated the effectiveness of BALs containing porcine primary hepatic spheroids as potential liver assistance components in a porcine hepatectomy model [9]. The proposed system improved animal survival up to 90 h, which was associated with an increased liver volume associated with signs of cell proliferation, demonstrated by Ki-67 positivity starting from 48 h post-hepatectomy. On the other hand, animals treated with standard medical therapies did not survive. 

Even though ALS devices and BAL systems are currently in clinical use and are continually being improved, their major function is to support normal liver function until the endogenous regenerative capacity of the liver reestablishes homeostasis, or until a donor organ becomes available. However, for patients with ESLD and acute liver failure (ALF), where endogenous regeneration is irreversibly impaired, partial replacement or implantation of healthy hepatic components is necessary to prolong the life of the patient. 

### 2.2. Cell Therapy

Transplantation of hepatocytes has been proposed as a non-invasive method to replace cells (chiefly hepatocytes) in a diseased liver. In theory, this is an attractive therapeutic alternative for orthotopic liver transplantation (OLT), as different sources of cells can be used, procedure-related complications are relatively rare, complex and expensive surgical procedures are not required, and repetitive transplantations might be possible [10]. During the last few decades, the potential of hepatocytes (primary or stem/progenitor-derived) to engraft and repopulate host livers has been thoroughly investigated in various animal models, as recently reviewed in detail [11]. Despite the encouraging results, none of the methods developed so far (i.e., cell sources, infusion routes) have proven to be robust enough for the repopulation of the host liver.

Nevertheless, hepatocyte transplantation has also moved into the clinic. However, this approach is only suitable for patients wherein the liver architecture has not been damaged due to long-standing fibrogenic responses, such as in metabolic disorder patients and rare patients with ALF [12]. Indeed, in ESLD following chronic liver injury, grafted hepatocytes encounter a very hostile environment which does not support engraftment and/or regeneration. Since the first clinical hepatocyte transplantation performed by Mito and Kusano in 1992, more than 100 patients have been reported to be treated by hepatocyte transplantation worldwide [12,13]. This has demonstrated that clinical hepatocyte transplantation can be safely performed. However, only modest increases in hepatic function have been reported to date, which has been mainly attributed to poor engraftment of the transplanted cells. The critical point in hepatocyte transplantation for inborn errors of metabolism is that transplanted hepatocytes should—theoretically—repopulate 5–10% of the recipient liver mass to overcome the failing function of hepatocytes. 

### 2.3. Three Dimensional Liver-like Constructs

Advancements in three-dimensional culture of hepatocytes (and non-parenchymal cells) in GMP-compatible environments expanded the effective use of cell therapy for ESLD or ALF patients. For instance, Dwahan et al. proposed alternative solutions for bleeding in coagulopathic patients upon portal venous injection of hepatocytes, as well as the need for long-term immunosuppression [14]. Specifically, primary human hepatocytes (PHHs) were encapsulated in alginate-based microcarrier beads before being transplanted intraperitoneally into pediatric ALF patients with acute liver failure. The hypothesis was that the presence of a bio-inert and semi-permeable barrier between the grafted PHHs and the host immune compartment would prevent immune rejection, while still allowing the secretion of vital soluble factors from the grafted PHHs until the endogenous regenerative process could occur. A similar technology previously demonstrated the regenerative potential of human pluripotent stem cell (hPSC)-derived hepatocyte-like cells (HLCs) in an immunocompetent mouse model, as shown by human albumin and α1-antitrypsin (A1AT) secretion in mouse serum [15]. 

Although the nature of the encapsulating material and the source of cells were shown to be determining factors in the success of implantation, controlling the spatial localization of hepatocytes and non-parenchymal cells (NPCs) within the encapsulating material may further improve this technology. Indeed, in the liver, hepatocytes are present in plates surrounded by sinusoidal endothelial cells and other NPCs in so-called sinusoids. Recreation of this spatial arrangement of the encapsulated cells might hence be beneficial. This could be accomplished by so-called 3D-bioprinting technologies. Three-dimensional-bioprinting technology enables precise positioning of cells within a hydrogel-based printing material, also termed “bioink”, prior to implantation. Kang et al. bioprinted mouse induced pluripotent stem cell (iPSC)-derived hepatocyte-like cells using alginate as bioink and implanted these constructs into the peritoneal cavity of NOD-SCID-γc^−/−^ mice. The implanted bioprinted constructs could be maintained in vivo up to 3 weeks, and, interestingly, higher albumin and lower alpha fetoprotein (AFP) transcript levels were identified in the in vivo grafted cells compared to their in vitro counterparts [16]. 

### 2.4. Shortcomings of the Aforementioned Technologies

As there are no effective medical therapies used to induce parenchymal regeneration in ESLD, parenchymal regeneration is, in general, not possible when the liver parenchyma has been damaged in fibrotic/cirrhotic liver, and because insufficient donor livers are available for liver transplantation, replacement of diseased livers with liver-simulating engineered products is a research area of utmost importance. Although the above-described possible therapies and bioengineering solutions are promising, they fail to recreate the anatomically complex make-up of the liver, wherein hepatocytes and NPCs surrounded by ECM are juxtaposed in liver sinusoids. Hence, investigators have started to focus on creating engineered products that mimic the liver architecture and vascular tree and contain liver-specific parenchymal (and non-parenchymal) cells to create grafts of clinically relevant size.

## 3. Emerging Concept: Whole-Organ Engineering

Whole-organ engineering is an emerging approach in the field of tissue engineering and regenerative medicine, aiming to generate ex vivo, clinically relevant, fully functional, and implantable organs. The novelty of this approach is that native organs from a xenogeneic source can be stripped of host cells via decellularization techniques, leaving the 3D extracellular matrix template of the donor organ. The remaining 3D scaffold contains tissue-specific ECM components, growth factors, as well as an intact vascular network and microarchitecture that can serve as an excellent environment for seeding and maintaining of organ-specific cells ex vivo [17]. 

Over the last decade, the generation of acellular templates via decellularization of various organs, including heart, pancreas, kidney, lung, and liver, has been reported [18,19,20,21]. Progress in decellularization technologies has made it possible to create organ-derived ECM constructs from the experimental to the clinical stage [22]. As the next challenge towards generating transplantable engineered organs, recellularization methods are being developed to generate transplantable bioengineered organs.

Here, we will present the evolution of organ decellularization and recellularization studies, focusing on the current state of the technology used for clinical applications. The primary challenges, such as potential immunogenicity, vascular and parenchymal functionality on the way to human transplantation trials, and the potential of stem/progenitor cells in generating functional grafts, are outlined. Strategies developed to overcome various limitations such as thrombogenicity, immunogenicity, and insufficient engraftment are discussed. 

### 3.1. Decellularized Liver Scaffolds

In each organ, the ECM composition and organization is unique and organ specific. The exact recreation of the ECM of a target organ de novo by means of in vitro synthetic techniques cannot yet be accomplished. This includes the recreation of both the specific mixture of ECM components, as well as the highly specific topographical nature of ECM. Liver ECM comprises less than 3% of the total area on a normal liver section, yet it includes various structural components such as collagens (e.g., type-I, -III, -IV and -V), elastin, fibronectin, and laminin in highly defined microarchitecture [23]. Decellularization applies a top-down approach to generate an acellular ECM-based scaffold of the target organ via removal of the native cellular content. The rationale behind decellularization is to remove the native cellular components through rigorous physical, chemical, and enzymatic treatments, while preserving the native ECM composition and architecture [24]. Significant progress has been made in generating decellularized organs from various species. Initial concerns raised about sterility and potential immunogenicity of acellular grafts have been extensively investigated and addressed. Currently, decellularization technologies have advanced to the point that allogeneic and xenogeneic acellular grafts can safely be used clinically. This progress towards (U.S. Food and Drug Administration) FDA-approved and clinically applicable acellular grafts from human and xenogeneic sources has been reviewed in detail in Parmaksiz et al. [22]. 

#### 3.1.1. Acellular Liver Scaffolds: Removal of the Endogenous Cellular Compartment

Acellular liver scaffolds have been prepared from rats, pigs, dogs, and humans, among others These were subsequently used to culture immortalized hepatic cell lines, primary rat or human hepatocytes, and stem cell-derived hepatocyte-like cells [25,26,27,28,29]. The relative effectiveness of various reagents such as sodium dodecyl sulfate (SDS), Triton X-100, peracetic acid, and sodium deoxycholate on decellularization of livers has been previously investigated [30,31]. Even though there is not yet a standardized liver decellularization procedure, a combination of trypsin with detergents such as Triton X-100 or SDS is predominantly applied. The lack of visible nuclear material in decellularized tissue sections (via DAPI, H&E staining) and near absence of double-stranded DNA (<50 ng/mg tissue) in the decellularized scaffold is commonly accepted as an indication of successful cell removal [32]. To estimate the extent of ECM preservation after decellularization, quantification of primary liver ECM components, such as collagen, elastin, glycosaminoglycans (GAGs), and growth factors has generally been applied. 

#### 3.1.2. Biomimetic Potential of Acellular Livers

In addition to the structural fibrillar elements such as collagens, liver ECM is composed of a unique array of growth factors and cytokines which are essential for liver homeostasis in vivo [33]. Decellularization protocols are optimized for the retention of such active components during cellular removal for in vitro culture of various liver cells. In the initial studies, decellularized liver scaffolds were used as hydrogels following solubilization of the ECM. Hydrogels generated from decellularized livers were used as 3D scaffolds for cell encapsulation, as an injectable material for in vivo healing, or as bioink for 3D bioprinting applications [25,26,27,28,29,34,35,36]. These studies indicated the bio-instructive potential of decellularized liver material for hepatic cells, and their ability to support the differentiation of progenitor cells towards hepatocytes and cells with an NPC fate [35]. Aleksander Skardal et al. demonstrated the presence of a wide range of growth factors and cytokines in decellularized porcine livers [26], including structural ECM components and high concentrations of important growth factors, such as basic fibroblast growth factor (bFGF), transforming growth factor-beta (TGFβ), epidermal growth factor (EGF), hepatocyte growth factor (HGF), and bone morphogenic proteins (BMP) 5 and 7. Improved functional characteristics of encapsulated PHHs, as measured by albumin production, urea secretion, and cytochrome p450 activity, were attributed to native biological cues provided in decellularized livers. In addition, retention of growth factors and cytokines that can induce differentiation of various progenitor cells towards hepatocyte- and/or non-parenchymal-like cells in vitro was also reported [35].

#### 3.1.3. Preserving the Vascular Tracts

Decellularization of the liver is accomplished by perfusion of decellularization reagents via the vascular tree, also preserving the tree-shaped network of arteries and veins. Subsequently, this intact vascular tract in the acellular organ can be used for seeding cells to repopulate the vascular and parenchymal liver compartments. Therefore, after successful engraftment of hepatocytes in the parenchymal compartment, the intact vascular network can be utilized to provide microcirculatory support for oxygen, nutrients, and growth factors to the engrafted cells, as well as removal of breakdown products. Moreover, the intact vasculature of the organ also allows the recellularized vasculature of the graft to be reconnected to the recipient circulatory system [37,38].

Perfusion decellularization and subsequent recellularization was first applied to cadaveric rat hearts by Ott et al. in 2008 [18]. They demonstrated that the detergent-based perfusion decellularization protocol successfully removed cardiomyocytes as well as endothelium from rat cadaveric hearts, as indicated by the absence of nuclei and cellular contractile elements, and significantly reduced DNA levels in the acellular graft. Multiple ECM proteins, including laminin, fibronectin, collagen I, and III, were retained in the graft, as well as an intact vascular network. After repopulating the decellularized graft with cardiomyocytes and endothelial cells, the recellularized heart redeveloped pump function by day 8, equivalent to about 2% of an intact adult rat heart.

The perfusion decellularization method has been adapted to generate whole acellular liver grafts from different organs. Kajbafzadeh et al. perfused sheep livers with decellularization reagents and demonstrated superior decellularization efficiency compared to simple immersion methods [39]. Subsequent approaches applying an oscillating flow rate of decellularization reagents in porcine and rat livers further improved the efficiency and homogeneity of cell removal. As an alternative, Pan et al. demonstrated successful in vivo decellularization of rat liver by generating bypass circulation through the portal vein and infrahepatic vena cava [40]. Furthermore, Gao et al. demonstrated that decellularized livers can also serve as an ex vivo platform to study embolization for both liquid and particle-based embolic agents, indicating that the vascular tracts of decellularized livers can also serve as a platform to study vascular dynamics in real time [41].

### 3.2. Repopulating Decellularized Liver Scaffolds

Creating a transplantable and functional bioengineered liver graft will require that the engrafted cells repopulate not only the parenchymal space, but also the macrovascular lining, sinusoids, and biliary tree. This requires (1) availability of the relevant types of cells for the repopulation of each cell compartment, (2) in vitro scaled-up cell production of cells in sufficient quantities for repopulation of all cell compartments, (3) and seeding and perfusion methods for maximal cell engraftment and repopulation. Notably, it is theoretically possible that only partial repopulation of the liver parenchyma would suffice, as even terminally differentiated hepatocytes can undergo cell division in vivo, as is observed upon liver damage or following partial hepatectomy. Hence, this could theoretically also occur when grafting a liver where only a fraction of the parenchyma contains hepatocytes [42]. In contrast, incomplete repopulation of the vascular bed would likely cause coagulation of the vascular tree when exposed to blood following transplantation into the recipient. Over the last decade, several proof-of-concept studies have been performed to evaluate the feasibility and current limitations of recellularization of liver grafts, chiefly in the livers of rodents. This has led to progressively improved seeding strategies used to recellularize livers. In the next few sections, we will review the cell sources used for liver recellularization, as well as different seeding strategies.

### 3.3. Parenchymal Repopulation

#### 3.3.1. Cell Types

##### Hepatoma Cell Lines

Hepatoblastoma cell lines, such as HepG2 cells, have been extensively used to recellularize the parenchymal space of acellular rat and porcine livers. The major advantages of HepG2 cells are their availability, long-term phenotypic stability, and high proliferative capacity [43]. This allows inexpensive in vitro expansion to produce cell numbers sufficient for liver recellularization. Several studies described that HepG2 cells efficiently engraft and remained viable and proliferative for up to 14 days following in vitro seeding in a perfused acellular liver [44,45,46,47]. However, HepG2 and other hepatoblastoma cells are not an option for clinical application due to their tumorigenicity and general inferior hepatocyte functionality compared to PHHs [11]. 

##### Primary Hepatocytes

Primary hepatocytes are clearly the best candidate cells for repopulation of the acellular liver parenchyma. Several studies have been published wherein primary hepatocytes were used to repopulate decellularized mouse or rat livers [40,41,48,49,50,51,52]. The rationale for these studies was to determine if acellular liver grafts can provide the required biological cues for infused primary hepatocytes to engraft efficiently and maintain essential functions following in vitro perfusion culture. When primary hepatocytes are cultured in 2D in classical culture dishes, they very quickly lose their mature hepatocellular phenotype and functionality, in part because of culture on stiff surfaces and lack of signals from the surrounding liver microenvironment [53,54]. However, Soto-Gutierrez et al. reported that when introduced in acellular rat liver grafts, inducible CYP1A2 activity and ammonia metabolism of primary murine hepatocytes could be maintained for up to a week [49]. Wu et al. compared the effectiveness of different decellularization methods of pig livers by seeding primary rat hepatocytes. The study concluded that Triton-SDS-Triton decellularization regime yields scaffolds with higher biomimetic potential, although still below the functionality levels which can be obtained by collagen sandwich cultures, though the reason for this was not explored [31]. When Yagi et al. grafted primary porcine hepatocytes in allogeneic acellular porcine liver grafts, they observed that nearly 50% of the grafted porcine hepatocytes were apoptotic by day 7, and that urea and albumin production decreased from day 2 after grafting onwards. Optimization of perfusion flow rate to adjust shear stress on engrafted cells, as well as potential co-culturing methods, have been raised as important parameters that should be established to enhance hepatocyte maintenance [55]. Debnath et al. showed that primary rat hepatocytes can repopulate decellularized Wistar rat livers and remain viable for 14 days. Although albumin secretion decreased from 3 days post seeding (following a similar trend as the 2D control cultured hepatocytes), engrafted cells showed expression of prominent lineage markers such as KRT19, ALB [52]. 

These models have provided insights into how to optimally seed primary hepatocytes in acellular liver grafts and have demonstrated that, at least in rat livers, hepatocytes remain alive and functional for 1 week. It is, however, not yet clear how to enable longer-term survival and stability of primary hepatocytes in acellular liver scaffolds.

#### 3.3.2. Why Stem/Progenitor Cells?

The primary rationale for using stem/progenitor-derived hepatocyte-like cells for repopulation studies is the proliferative capacity and plasticity of progenitor cells found in liver. In a healthy liver, proliferation of mature hepatocytes is responsible for the regeneration of the parenchyma. However, severe liver damage surpasses the regenerative capacity of mature hepatocytes, and as a result, activates quiescent hepatic progenitor cells (HPCs) for the reestablishment of parenchymal homeostasis [56]. Although the mechanism by which HPCs contribute to liver regeneration is not yet fully understood, their ability to proliferate and differentiate towards mature hepatocytes and/or biliary cells is reported to be crucial for liver regeneration [57]. The second rationale is that the isolation of human non-parenchymal liver cells is challenging partly due to lack of reliable markers (e.g., stellate cells), and in vitro expansion of isolated cells, while preserving their cell-specific characteristics is difficult. Therefore, stem or progenitor cell-derived non-parenchymal cells potentially circumvents the difficulties related to isolation and mass expansion in vitro.

It is also possible to generate HPCs from extrahepatic sources such as Mesenchymal stem cells (MSCs), or human embryonic and induced pluripotent stem cells (hESCs and hiPSCs) [11]. Stem-cell-derived HPCs differentiate into cells termed “hepatocyte-like cells (HLCs)”, as they do not fully reconstitute all features of mature hepatocytes. Hepatoblasts, differentiated from, for instance, pluripotent stem cells (PSCs), express markers similar to HPCs, such as α-fetoprotein (AFP) and keratin 19 (KRT19), and may therefore be good candidates for repopulation of decellularized livers. We demonstrated that PSC progeny harvested at the HPC stage (on day 8 of differentiation) yielded superior differentiation efficiency in synthetic matrixes compared with cells harvested at earlier or later stages of hepatic differentiation, supporting the idea of infusing PSC-derived HPCs for repopulation studies. [58] Other studies have also used (i) PSC-derived HPCs for decellularized liver repopulation, as will be discussed in detail in the following sections.

##### Fetal Liver Cells

Several groups used murine or human fetal HPCs for recellularization studies. Ogiso et al. compared the engraftment and repopulation ability of adult or E14.5 fetal murine hepatocytes in acellular rat liver scaffolds. Both cell types engrafted efficiently when infused via the biliary ducts, giving rise to an approximately 80% initial engraftment. Two days post-infusion, the percentage of Ki-67 positive cells was significantly higher in acellular livers seeded with fetal rather than postnatal hepatocytes; and consequently, repopulation was significantly higher following infusion of fetal compared with postnatal hepatocytes [59]. To test if fetal HPCs retain their proliferative potential after transplantation, Wang et al., [60] infused E13.5–E14.5 immortalized mouse HPCs overexpressing human EGF or GFP in decellularized mouse livers. After 24 h of perfusion culture, recellularized scaffolds were implanted into the kidney capsule of athymic nude mice and were maintained for the next 10 days. Cells overexpressing human EGF showed a greater survival and proliferation rate compared to GFP expressing control cells. The study emphasized that human EGF plays a significant role in the engraftment and survival of infused cells in decellularized liver grafts. Similar approaches might be implemented to identify additional growth factors that may enhance engraftment efficiency and survival of infused cells. 

Similar studies using human fetal HPCs have also been conducted. In these studies, the cells were co-infused with human fetal hepatic stellate cells (HSCs) or Human Umbilical Vascular Endothelial Cells (HUVECs); therefore, these studies will be discussed in the co-seeding section.

##### Liver-Derived Stem Cells

Adult liver stem cells, identified by expression of Leucine-rich repeat-containing G 5 (LGR-5), can be culture expanded using organoid cultures [61,62]. Although these cells have not yet been used in liver repopulation studies, they might be an interesting source of cells, as they have very high proliferative capacity and bipotential differentiation ability towards hepatocytes and biliary cells. Hence, in vitro mass expansion might be used to provide an autologous (or allogeneic) cell source for repopulation of the hepatocyte and biliary cell compartment of acellular liver scaffolds [63].

##### Human Induced Pluripotent Stem Cells

Induced pluripotent stem cells (iPSC) can be generated from any nucleated cell via cellular reprogramming, and subsequently redifferentiated into hepatocyte-like cells. The major advantage of hiPSCs is that they have very extensive self-renewal ability, enabling the creation of very large cell doses that can be subsequently differentiated in sufficient HLCs for recellularization of a human-size acellular liver graft. Minami et al. generated hepatocyte-like cells from hiPSCs and infused day 20 progeny into decellularized rat livers via the biliary duct [64]. Two days after perfusion culture, engrafted cells stained positive for albumin, AFP, and CYP3A4. Moreover, *CYP3A4* mRNA levels were higher than those measured in 2D control cultures. However, despite the apparent more mature profile of cells in the decellularized liver graft, the cumulative albumin levels in culture supernatants were significantly lower in liver grafts compared to 2D control cultures. More recently, Acun et al. seeded decellularized rat liver scaffolds with undifferentiated iPSCs and proceeded differentiation to commit hepatic lineage during perfusion culture. Exposure to unique composition and microarchitecture of the native liver matrix was shown to generate more mature hepatocyte-like cells after a week, compared to iPSCs differentiated in 3D Geltrex hydrogels [65]. Activation of Wnt/β-catenin pathway has been also shown in iPSCs seeded in decellularized rat livers committed to hepatic lineage [66].

It should, however, be kept in mind that currently, as is true for many other iPSC progeny, hiPSC-derived HLCs are not fully mature [67]. Moreover, persisting undifferentiated PSCs may form teratomas [68]. A potential plus point for iPSC-derived progeny is that iPSC could be generated in an autologous manner, eliminating the need for immunosuppressive treatment following transplantation. However, the time required for generating autologous iPSCs, iPSC expansion, differentiation towards HLCs, and quality assessment before it becomes possible to graft the cells in acellular liver grafts may surpass the period wherein a novel organ is required to support/treat ESLD patients. In such a scenario, transplantation-ready livers repopulated with allogeneic iPSC-HLCs could be considered, even if recipients would then require immunosuppressive therapy.

##### Mesenchymal Stem Cells

Human Mesenchymal Stem Cells (hMSCs) derived from various sources, including bone marrow and adipose tissue, can support liver function and regeneration without prior hepatic commitment [11]. However, it is generally accepted that the main mechanism for the therapeutic effect of MSCs is trophic support of the endogenous liver, enhancing parenchymal regeneration via the prevention of apoptosis, stimulation of hepatocyte proliferation and angiogenesis, and activation of resident stem cells.

There are, however, also reports indicating that MSCs might be used to repopulate acellular livers. Jiang et al. infused decellularized mouse livers with mouse bone marrow-derived MSCs and then induced hepatic commitment following engraftment [69]. Compared to differentiation in 2D cultures, the decellularized liver microenvironment enhanced the expression of hepatocyte specific markers at the mRNA (i.e., *HNF-1/4A, -1B*) and protein (Albumin, CK19) levels. Furthermore, following grafting of CCL4 treated mice with acellular liver graft cultured MSCs, slight improved survival of mice (2 of 6 surviving) was seen compared with placebo-treated mice (zero of six surviving) or mice infused with MSCs (one of six surviving). In another study, porcine adipose-derived mesenchymal stem cells were seeded on decellularized porcine liver pieces (without perfusion). Although the viability of seeded cells confirmed after 10 days in vitro static culture was demonstrated, assessment of the potential induction of hepatic commitment in the acellular grafts was not tested [70].

##### Conclusions on Different Cell Types

Overall, we believe that MSCs are valuable when studying the biomimetic potential of decellularized liver scaffolds. In particular, the contribution of the unperturbed ECM architecture, which can only be achieved via perfusion decellularization, would be ideal for studying the possible hepatic differentiation of engrafted MSCs. However, as the final functionality of repopulated liver grafts is highly dependent on the hepatic differentiation of engrafted cells, liver stem cells, HPCs, or iPSC-HLCs (and/or non-parenchymal cells), are likely much better candidates. The trophic effects of co-infused MSCs might be exploited to enhance the repopulation of hepatic cells, and future studies could then investigate synergistic effect of co-infused MSCs with hepatocytes and/or non-parenchymal cells.

#### 3.3.3. Enhancing Parenchymal Engraftment Efficiency

As the number of hepatocytes required for recellularization of clinically relevant sized organs is very high, [71] in vitro cell expansion methods are being evaluated, with limited success [72]. Therefore, other means for enhancing recellularization efficiency have been investigated. 

The liver is a highly structured organ, wherein capillary sinusoids are located between hepatic cell plates that are connected to the hepatic artery and portal vein (portal tract) and the hepatic vein (venous tract). Moreover, bile produced by hepatocytes is removed via biliary canals which are also located in the portal zone. These vessels/tracts allow the effective distribution of oxygen and nutrients throughout the hepatic lobule, and removal of breakdown products via the biliary tract and hepatic vein, respectively [1]. The extensive branching of these different intrahepatic canals and vessel tracts following decellularization has been exploited for seeding parenchymal (and non-parenchymal) cells into acellular livers. In the next section, we will discuss how utilizing the well-branched vasculature and/or biliary canals, infusing cells in a multistep fashion, vs. direct intraparenchymal injection may affect parenchymal recellularization.

##### Infusion Routes and Dynamic Culture

The portal vein has been extensively used as a seeding route for hepatocytes, due to its accessibility, wide lumen, and extensive branching. As an alternative to the portal tract, Ogiso et al. used the biliary canals, which are more narrow and less branched, to infuse parenchymal cells into acellular livers [59]. They demonstrated that 3 h after infusion, the number of hepatocytes seeded via the biliary duct repopulated the parenchymal space approximately three-fold more efficiently than those seeded via the portal vein. In addition, a large proportion of hepatocytes seeded via the portal vein remained inside the portal lumen, which would be detrimental when grafting the organ in vivo, as this would interfere with blood flow, and may also preclude the subsequent efficient seeding and coverage of the vascular tree with endothelial cells. Sassi et al. showed the necessity of continuous unidirectional media flow (i.e., perfusion culture) for the maintenance of engrafted cells [73]. Higher viability, distribution, and function of luciferase-expressing HepG2 (Luc^+^HepG2) cells engrafted in decellularized rat livers was observed in perfusion culture in comparison with static cultures for up to 11 days.

##### Multi-Step Infusion

To avoid large numbers of hepatocytes remaining in the vascular tree after seeding, and hence block the vasculature, different intravascular injection and/or perfusion schedules have been investigated. For instance, Soto-Gutierrez et al. demonstrated that a multistep infusion of cells via the portal vein not only increased the parenchymal repopulation rate, but also enhanced the functionality of engrafted cells, as assessed by albumin production, CYP1A1/2 activity, and ammonia removal. They hypothesized that the improved repopulation and functionality of the cells grafted via this multistep infusion protocol might be due to the different mechanical stresses applied [49].

##### Direct Parenchymal Injection

To address whether direct delivery of hepatocytes in the parenchymal space might increase repopulation, as it does not require that seeded cells infiltrate from the vessel tracts into the parenchyma, Zhou et al. compared the repopulation efficiency of direct multilocational parenchymal injection versus portal vein infusion of the buffalo rat liver (BRL) epithelial-like cell line [74]. Cells delivered via parenchymal injection engrafted more efficiently, possibly because a portion of the cells in the portal lumen was washed away. Grafting directly into the parenchyma resulted in increased albumin secretion compared with 2D control cultures. 

##### Conclusions on Seeding Methods

The above studies suggest that utilizing the biliary tree for hepatocyte engraftment might be superior to the use of the vascular tree. In addition, direct parenchymal injection may also enhance parenchymal repopulation. However, this is a more invasive method to deliver parenchymal cells, as needle injection might locally disrupt the capillaries at the injection site. Utilizing different vascular tracts of the liver scaffolds by applying sequential seeding might also enhance parenchymal engraftment efficiency. As transplantation of recellularized liver graft will also require that the endothelial lining is repopulated (discussed in the following section in detail), the infusion route for hepatocytes or HLCs should be carefully selected. The approach should ascertain that the lumen of the main vascular tracts, e.g., the portal and central vein, and hepatic arteries, as well as sinusoidal tracts, remain accessible for grafting of endothelial cells. 

### 3.4. Vascular Regeneration: Re-Lining the Endothelium

Effective repopulation of the parenchyma with hepatocytes, HPCs, or HLCs is essential for the liver functionality of the recellularized liver graft. Yet, an equally important criteria to generate a transplantable recellularized organ is the repopulation of the vasculature, such that the organ can be connected to the endogenous vasculature and endogenous vessels remain patent.

#### 3.4.1. Endothelium in Liver

The vasculature of an organ is required for delivering nutrients to and removing breakdown products from the organ. The lumen of the vascular tree in the liver is covered with a continuous lining of endothelial cells. This endothelial barrier prevents blood contact with the underlying ECM components. Upon disruption of endothelial continuity, the sub-endothelial matrix is exposed in the vascular lumen. Consequently, circulating platelets adhere to the exposed ECM via integrins, primarily mediated by the vascular wall-associated von Willebrand factor [75,76]. Although this cascade of events is a defense mechanism used to stop bleeding caused by injury, excessive platelet deposition in the vascular lumen causes thrombosis and impedes normal blood flow. Collagen fibers are abundantly exposed in the lumen of large vascular tracts in decellularized livers, creating an excellent substrate for platelet deposition. On the other hand, they serve as the substrate to which endothelial cells can attach via an integrin-mediated process [77]. Therefore, to be able to graft a recellularized organ in vivo and prevent blood coagulation, endothelialization of the vasculature is required. To accomplish this, endothelial cells are perfused through the venous or arterial tract of the decellularized grafts. Prevention of thrombosis upon transplantation of recellularized liver grafts essentially depends on the near complete re-endothelialization of the vascular tree [78]. Studies wherein endothelial cells are grafted combined with other strategies for revascularization are outlined in the following section.

#### 3.4.2. Cell Types

##### Endothelial Cell Lines

Both murine and human endothelial cell lines have been used to recellularize the vascular tract of decellularized liver scaffolds [46,79]. Ko et al. used murine MS1 endothelial cells to recellularize the vascular tract of decellularized pig scaffolds, as a first large animal transplantation model [79]. These revascularized scaffolds were capable of supporting flow up to 24 h post transplantation, as shown by fluoroscopic angiography and Doppler ultrasound. HUVEC-derived immortalized endothelial cell lines were also shown to support physiological blood flow after transplantation in a porcine model [46]. Contrast radiography showed a clear branching pattern in the endothelialized liver scaffold 24 h after transplantation into recipient pigs, indicating blood barrier function of endothelialized vascular lumens. In both studies, the vascular lumen of decellularized pig liver scaffolds has been modified to enhance cell attachment, which will be discussed in detail in the following chapter.

##### Primary Endothelial Cells

For human implementation, recellularized liver grafts essentially require endothelial cells from human origin. To prevent acute graft failure upon transplantation, the lumen of the vascular tract of the decellularized liver needs to be covered to prevent blood coagulation by macrovascular endothelial cells. Longer-term unobstructed blood flow also requires the lining of sinusoidal tracts with liver sinusoidal endothelial cells (LSECs).

##### Macrovascular Endothelial Cells

Methods for the isolation, expansion, and maintenance of non-transformed HUVECs have been well established. Various groups used HUVECs to cover the lumen of the macrovascular lining of acellular rat, porcine, and human livers using slightly different strategies [80,81,82,83]. Watanabe et al. [83] tested different flow rates on the angiogenic behavior of HUVECs engrafted in decellularized rat livers. They demonstrated that the properties of both macrovascular and sinusoid-scale tracts, as well as the orientation of HUVECs in the vascular lumen, were highly dependent on the applied flow rate. Although these results obtained using a decellularized rat liver model set the stage for re-endothelialization of liver grafts, testing these parameters in human-sized livers, such as those of pigs, is of importance. Shaheen et al. [84], demonstrated that HUVECs, which initially attached to the macrovascular lumen, also repopulated the capillary bed within the parenchyma of decellularized pig livers. Interestingly, this was associated with an apparent switch from CD31+ macrovascular endothelial cells to cells with a more LSEC-like phenotype, characterized by the presence of LYVE1, indicating that cues for LSEC formation might be retained in the avascular graft vascular tree.

##### Liver Sinusoidal Endothelial Cells

The endothelialization of parenchymal sinusoids is of equal importance to covering the macrovascular lumen, given that they form a barrier between sinusoidal blood and hepatocytes. This is particularly important if the parenchyma of transplanted reendothelialized liver grafts is also repopulated with hepatocytes. Direct contact between hepatocytes and blood is known to cause an instant blood-mediated inflammatory reaction (IBMIR), in which inflammatory cells, including monocytes, granulocytes, and natural killer (NK) cells, are activated and cause inflammation. The occurrence of IBMIR can be prevented by an endothelial lining of the sinusoids in decellularized liver scaffolds. However, difficulties related to isolation and large-scale in vitro expansion of primary LSECs hamper their use in liver recellularization studies [85]. 

##### Stem/Progenitor Cell-Derived Endothelial-like Cells

Generation of endothelial-like cells from hiPSCs has been described using various differentiation protocols [86]. In addition, some studies showed that it might be possible to transform such iPsc endothelial cells to an LSEC phenotype via modification, TGFβ pathway inhibition, or genetic engineering [87,88,89]. These hiPSC-derived LSECs may be good candidates to cover the sinusoidal endothelial lining of the liver parenchyma. However, as Shaheen et al. found that HUVECs gain the expression of LYVE-1 when present in sinusoids in the liver parenchyma [84], it is also possible that grafting hiPSC-derived endothelial cells not previously connected to LSECs might undergo a phenotype shift from a macrovascular- to a sinusoidal endothelial-like phenotype when they engraft in liver sinusoids. 

From the perspective of whole organ recellularization studies, the minimal endothelial cell requirements would be that they can cover the lumen of the vascular tract, show angiogenic behavior, and maintain viability on the long term. Repopulating the vascular tract mainly depends on initial engraftment following perfusion and subsequent proliferation to cover the collagen-rich basement of the large vessel vascular lumen. Angiogenic behavior would be required for the migration of engrafted endothelial-like cells through the parenchymal space to line sinusoids. Hence, the development of iPSC-derived endothelial-like cells should initially consider these aspects to enable the generation of a transplantable organ with a functional vasculature. 

Endothelialization of rat livers by hiPSC-derived endothelial cells was first described by Takeishi et al., and will be discussed in the Co-seeding and Co-culture section [90]. To our knowledge, recellularization of large animal organ models by hiPSC-derived endothelial cells has not yet been reported. 

#### 3.4.3. Vascular Lumen Modifications

Multiple approaches have been developed to enhance the attachment of endothelial cells to the vascular ECM, or to enhance vascular patency following transplantation of the graft in vivo, which are summarized below. 

##### Antibody Coating

Platelet and endothelial cell adhesion molecule 1 (PECAM-1), also known as the CD31 antigen, involved in cell–cell adhesion via cell junctions, is expressed extensively on large vessel endothelial cells [91]. Therefore, Ko et al. investigated the effectiveness of antibody conjugation-mediated cell attachment [79]. The vascular lumen of acellular rat liver was coated by perfusion with an anti-mouse CD31 antibody through the hepatic artery, portal vein, and vena cava via covalent attachment. Subsequent infusion of mouse MS1 endothelial cells resulted in a significantly larger number of engrafted cells in both large and smaller vessels (<200 µm) of the antibody-treated compared to untreated liver scaffolds. Moreover, platelet adhesion was observed to be significantly lower in both portal and central veins of antibody-coated recellularized livers, indicating an enhanced vascular patency.

##### Protein or Peptide Conjugation

Other groups tested covalent modification of acellular lumens using endothelial adhesive constructs such as full-length fibrinogen and fibrinogen-mimicking REDV peptide to provide integrin-mediated endothelial cell adhesion. Compared with antibody coating, these methods would provide an inexpensive way of making acellular lumens more supportive for seeded endothelial cells. Devalliere et al. fused the cell-adhesive REDV peptide to an elastin-like peptide (ELP), and coated the lumen of rat decellularized scaffold vasculature via N-ethyl-N′-(3-(dimethylamino)propyl)carbodiimide/N-hydroxysuccinimide (EDC/NHS) chemistry [92]. When coated and uncoated grafts were seeded with the EA.hy926 endothelial cell line, the engraftment rates did not differ. However, more vessels were clogged by unattached endothelial cells in untreated grafts. Endothelium in REDV-treated liver scaffolds expressed significantly more *eNOS, VEGF,* and *VE-cadherin* mRNA four days after grafting, which was associated with decreased platelet adhesion after platelet-rich plasma perfusion. Similarly, pre-incubation with full-length fibrinogen enhanced engraftment of HUVECs not only engrafted in large vessels, but also sinusoid-scale microvessels [83]. 

##### Vascular Heparinization

Although endothelialization of decellularized liver grafts increases the vascular patency significantly, achieving a continuous endothelial lining throughout the organ remains challenging. Vascular heparinization has therefore been suggested by several groups to mask the prothrombotic properties of collagen. Due to its anticoagulant properties, heparin is extensively and successfully used to insert blood-contacting prostheses [93]. Heparin coating of decellularized liver vascular lumens can be achieved via a layer-by-layer electrostatic immobilization process, or through covalent conjugation via EDC-NHS chemistry. The former has been used by Bruinsma et al. to modify the vascular lumen of rat liver scaffolds [94]. Heparinized acellular liver scaffolds showed minimal circulatory obstruction during a two-hour-long ex vivo diluted blood circulation, while visible blood clots were observed throughout the unmodified scaffolds. Although ex vivo blood circulation was feasible, the heparinized liver graft failed to prevent thrombosis in the setting of heterotopic transplantation. A similar strategy was also applied to decellularized porcine livers. However, aside from electrostatic heparin immobilization, heparin was also covalently conjugated to the vascular lumen of porcine liver grafts [95]. When transplanted into recipient pigs, massive thrombus formation was observed in the portal tract of unmodified grafts within twenty minutes, while no visible signs of thrombosis were observed in covalently and electrostatically heparinized livers for one hour. Nevertheless, blood flow decreased progressively from inflow to outflow after one hour in heparinized livers. The absence of continuous endothelium allowed blood leakage into the parenchyma, and hence obstructed blood flow throughout her scaffold, starting from the sinusoids. Thus, even if heparinization might prevent acute graft failure due to platelet deposition, re-endothelialization is essential for long-term functioning of the recellularized scaffolds. 

##### Conclusions on Vascular Modifications

In conclusion, heparinization of the vascular lumen of decellularized liver scaffolds can prevent coagulation of blood due to interaction with the naked ECM in the short term. However, a continuous endothelial lining will be required to prevent coagulation and to allow the fabricated liver to connect to the circulation in vivo. Therefore, combining endothelialization and heparinization is likely a way forward to enhance vascular patency of in vivo grafted decellularized liver grafts.

## 4. Co-Seeding and Co-Culture of Parenchymal and Non-Parenchymal Components

The overall functionality of the liver in vivo relies on the activity and intercommunication of different cell types, including hepatocytes, macrovascular endothelial cells, sinusoidal endothelial cells, stellate cells, and pericytes (in the liver, also termed hepatic stellate cells). Ideally, repopulated acellular liver grafts should include parenchymal as well as all NPCs in their anatomically correct location throughout the liver lobule. For instance, ourselves and other researchers demonstrated that hepatocytes, either of primary or stem-cell origin, cultured with NPCs attain a more mature phenotype and functionality [58,96,97]. Based on these findings, we believe co-seeding and co-culture of hepatocytes and NPCs will improve the functionality of the repopulated liver grafts. 

Co-seeding and co-culture of hepatocytes from various sources with different NPCs in decellularized liver scaffolds has been studied. Hussein et al. [46] assessed the synergistic effect of co-seeded HepG2 cells and the immortalized HUVEC cell line, EA.hy926, in acellular porcine liver scaffolds. Co-seeding was achieved by first repopulating the heparinized liver parenchymal space with HepG2 cells via portal infusion, and then perfusing EA.hy926 cells through both the portal vein and hepatic artery, which were also coated with a heparin–gelatin mixture. After ten days of ex vivo perfusion coculture, RT-PCR demonstrated higher levels of *ALBUMIN, CYP3A4,* and *GPX1* in scaffolds grafted with both HepG2 and EA.hy926 compared with scaffolds repopulated with HepG2 cells. Moreover, albumin and urea levels in the perfusate medium collected from endothelium/HepG2 grafts were higher than HepG2-only seeded grafts. Hassanein et al. [98] seeded HepG2 cells via the biliary tract in decellularized rat liver scaffolds and HUVECs via the portal vein. They demonstrated that successful engraftment of both the vascular and parenchymal compartments was obtained, even if the effect on hepatic cell function was not addressed. Thanapirom et al. evaluated the co-seeding and co-culture of HepG2 and the human hepatic stellate cell line (i.e., LX2) in healthy or cirrhotic decellularized human livers as a potential drug-testing platform [47]. Following engraftment and 13 days of perfusion co-culture, repopulated livers showed upregulation of fibrotic markers in response to TGFβ. Interestingly, cells co-cultured in decellularized cirrhotic livers showed greater fibrotic response upon TGFβ induction compared to healthy decellularized liver counterparts. This indicates that the distorted liver ECM microenvironment creates a pre-disposition to fibrotic response, and potentially other distortions as well, such as inflammation.

Kojima et al. [99] co-seeded endothelial cells, more specifically rat-liver-derived LSECs and rat hepatocytes, via the portal vein and biliary tract, respectively. They too demonstrated successful vascularization of the vascular and parenchymal compartments. Anderson et al. demonstrated that it is also possible to repopulate a porcine graft, first endothelialized with HUVECs by seeding via both portal vein and infrahepatic inferior vena cava, and after 14 days of reendothelialization process, with primary porcine hepatocytes infused via the biliary tract [100]. This sequential seeding of a large animal model graft successfully repopulated the parenchymal compartment once the endothelial compartment had been repopulated. Importantly, the above studies also demonstrated the beneficial effect of co-seeding of the endothelial and parenchymal compartment on the functionality of the hepatocytes, as shown by albumin and urea production, and ammonia clearance. 

Kupffer cells (i.e., liver-resident macrophages) and monocyte-derived macrophages account for approximately 15% of the total healthy liver population. They are directly involved in elimination of endotoxins and degenerated liver cells; and (2) regulation of the innate immune response and host defense in response to blood-borne infections [101]. Kupffer cells are, at least in mouse, derived from primitive hematopoietic stem cells in the yolk sac, and remain in the liver for life [102]. However, studies in mouse have demonstrated that macrophages derived from adult hematopoietic stem cells can replace Kupffer cells when the resident Kupffer cells are eliminated [102]. Hence, one may surmise that when the recellularized liver is grafted in vivo, macrophages from the adult hematopoietic system may repopulate the liver and acquire properties of Kupffer cells. Therefore, efficient repopulation of decellularized livers primarily with hepatocyte (-like) cells, and then recreating intact endothelium with endothelial (-like) or stellate/pericyte (-like) cells, which should recreate the Kupffer cell niche, might suffice.

### Stem/Progenitor-Derived Cells

As already alluded to above, pluripotent stem cells that can be expanded massively may serve as a good cell source for liver repopulation, as they cannot only be fated to HLCs, but should also be capable of differentiation into all NPCs. The latter included cholangiocyte-, endothelium- and stellate-like cells [89,103,104] that could be used to repopulate liver scaffolds. Takeishi et al. provided a proof of principle by generating not only hepatocyte-like cells, but also cholangiocyte- and endothelial-like cells from hiPSCs, which were co-seeded together with primary human liver (not iPSC)-derived MSCs and fibroblasts into decellularized rat livers [90]. They accomplished a near 80% repopulation of the vascular bed and 65% and 70% of the biliary and parenchymal compartment, which was associated with secretion of liver-related compounds such as bile acids, urea, and human A1AT, ex vivo. Moreover, the repopulated graft was shown to be viable for 4 days upon auxiliary transplantation. Therefore, this small animal recellularization model provides evidence that iPSC-derived parenchymal and non-parenchymal cells may be suitable for repopulation of grafts prior to implantation in vivo.

## 5. Essential Steps Prior to Transplantation: Assessing Vascular Patency

From a clinical standpoint, it is imperative that one can predict prior to transplantation whether a graft may fail. This may be feasible by exploiting ex vivo blood perfusion systems [48,84,92]. Such perfusion systems were originally developed to avoid cold ischemia-induced hypoxia of non-perfused donor livers. The normothermic perfusion of nutrients and therapeutic cocktails, including insulin and bile flow promoters, at physiological pH increases the cellular viability of the donor organ [105]. In addition to allowing for longer transport times and preserving organ quality, the system has also been used to assess allograft function prior to transplantation. The perfusion of freshly isolated blood from the host species through the endothelialized liver graft provides a surrogate model of transplantation and allows quantification of platelet deposition after blood perfusion by intensity of integrin-αIIβ staining, enumeration of inflow versus outflow platelet number, radiography, and Doppler ultrasonography [38,48,80,84,94,95,99]. The main advantage of evaluating re-endothelialized liver grafts in an ex vivo blood circulation system is that various recellularization parameters, including the type of cells, the seeding number, and route, and the duration of static and perfusion culture, all affecting vascular patency, can be effectively tested before grafting the organ in vivo. Such an initial screen reduces the number of recipient animals required for transplantation experiments and allows for a mechanistic investigation of the hemocompatibility of engrafted cells. Although ex vivo recipient blood circulation has been widely shown as predictive for vascular patency of recellularized liver grafts, Bruinsma et al. [94] reported that even if ex vivo rat blood circulation could predict the thrombogenic potential of recellularized rat liver, it could not predict blood congestion leading to obstructed normal flow observed in all grafts 24 h post-transplantation. Those findings indicate that even though ex vivo circulation systems can effectively predict early graft failure, they might fail to fully mimic in vivo conditions.

## 6. Current Status of Transplantation Studies

### 6.1. Rodent Models

Ex vivo blood circulation systems allow us to evaluate the continuity and integrity of the endothelial lining of recellularized liver. However, transplantation studies are an essential part of evaluating the applicability of recellularized livers towards clinical applications. Besides testing the vascular patency in more biologically relevant systems, transplantation studies are intended to test a potential inflammatory and/or immune response from the host towards the graft. For this purpose, rodent models have been used extensively. Rodents are preferred over larger animal models as they are easy to maintain, less costly, ethically more acceptable, and above all, immune incompatibility can be readily modeled. Hence, aside from allowing testing of numerous parameters that are important for the successful creation and grafting of recellularized livers, rodent models also allow mechanistic answers in regard to the role of immunological rejection. Several studies reported grafting of acellular rodent livers, recellularized with various types of rodent-derived cells, into allogenic rodents (Table 1) [48,50,60,69,94]. 

While rodent models serve as a convenient platform to answer initial research questions about vascular patency and graft immunogenicity, rodent models cannot be the sole model used before clinical application due to size and anatomical differences.

### 6.2. Large Animal Models

To ultimately be able to graft recellularized livers in patients will require that liver scaffolds of comparable size, anatomy, and physiology to their human counterparts are grafted in vivo. As human livers are not readily available for decellularization and recellularization processes, large animal model livers have been used. Porcine liver scaffolds are preferred, as they are relatively easy to obtain, they resemble human livers well, and there is ample experience grafting the (recellularized) livers back into porcine models. To determine the potential thrombogenicity and immune response between the graft and the recipient animal, as well as possible surgical complications, auxiliary transplantation models have been suggested [106]. Partial hepatectomy and nephrectomy provides space for liver grafts and allows the graft to be connected to the host circulatory system. End-to-side anastomose between the graft and recipient animal portal vein and inferior vena cava can be used to supply and drain blood, respectively. The risk of clot formation following grafting can be managed by postoperative antiplatelet or anticoagulation therapies. Graft rejection can be alleviated by the administration of immunosuppressants and/or removal of the recipient spleen prior to transplantation. Over the last decade, several groups tested these essential parameters (Table 2) [70,79,84,107]. Ko et al. [79] tested the vascular patency of acellular porcine livers endothelialized by the murine endothelial cell line (MS1) after heterotopic transplantation in Yorkshire pigs. This revealed that endothelialization is crucial for the initial compatibility of liver grafts, as non-endothelialized livers caused adherence of porcine platelets to the lumen of both portal and central veins. 

Within the grafts, comparable blood inflow and outflow rate for 24 h was observed in the portal vein and inferior vena cava, respectively. Shaheen et al. [84] demonstrated that immunosuppression following transplantation extended the patency of the vascular tree of HUVEC engrafted liver grafts from 3.5 to 15 days. However, following withdrawal of immunosuppression, graft thrombosis was seen, indicating that immunological responses contributed to thrombosis and graft loss. In the following study, Anderson et al. first revascularized decellularized porcine livers with HUVECs, and then seeded primary pig hepatocytes to generate a graft with active parenchyma and patent vasculature. Repopulated liver scaffolds showed liver-specific functions such as albumin production, ammonia detoxification, and urea synthesis. Additionally, repopulated livers were used in a heterotopic large animal implantation model, wherein the implanted grafts survived in vivo up to 48 h. 

## 7. Challenges towards Clinics

Numerous challenges stand between the current progress of whole-organ engineering and its potential clinical application. The selection of the acellular scaffold will likely result in an acellular pig scaffold, which are the most suitable, as they resemble the human liver well [108]. However, the challenge lies in (1) the source of cells to be used; (2) in ensuring that the endothelial lining of large as well as small blood vessels is completely repopulated with endothelial cells and does not induce blood clothing or blood flow obstruction; and ensuring that (3) the liver parenchymal cells, with high functionality, repopulate the liver parenchyma. It is less clear whether all other liver cells such as biliary cells and hepatic stellate need to be incorporated, or whether they could be derived from the hepatic cell population (e.g., when hepatoblasts are used) and/or can be derived from the host. 

### 7.1. Source of Cells

Although allogeneic primary liver-derived hepatocytes and large vessels, as well as liver sinusoidal endothelial cells, would be the ideal cell source, this is not feasible. First, the cell source is scarce, and when cultured ex vivo, both cell types cannot be readily expanded and very quickly lose their mature functions. Recent advancements in iPSC technology now allow for the differentiation of hepatocyte- and non-parenchymal-like cells from hiPSCs, and hence may be a valid alternative for primary liver-derived cells. However, iPSC-derived progeny still did not achieve the maturation levels of primary liver-derived cells.

The advantage of iPSC-derived liver cells from autologous cell sources would potentially eliminate the need for immunosuppressive therapy following transplantation. Nevertheless, the time required for generation of iPSC lines, iPSC expansion, and differentiation towards different cell types might surpass the expected lifetime of the patient. Therefore, an alternative could be readily available allogeneic stem-cell sources, even if this will then require administration of immunosuppression. In addition, creating robust methods for large-scale expansion and subsequent differentiation of stem cells under GMP conditions and in an economical way will be required to create clinically relevant cells. Currently, progress is being made towards clinical-scale and -grade stem cell expansion and differentiation [109]. 

### 7.2. Repopulation of Vasculature and Parenchymal Compartments

A number of studies reviewed in this manuscript reported at least partial recellularization of acellular liver scaffolds by hepatocytes and/or endothelial cells, and various strategies to enhance their engraftment and survival. However, for large animal liver scaffolds, full and long-term stable repopulation remains a challenge. Different approaches, such as enhancing the attachment of endothelial cells to the vessel tract, are being developed [78], which may make full re-endothelialization possible. Likewise, different approaches are being tested that may support the aim to repopulate the parenchyma to a sufficient level for the support of liver function following grafting, including the use of hepatic progenitors rather than mature hepatocytes, and this combined with methods to enhance repopulation, e.g., by growth factors that enhance proliferation or also maturation [64]. It remains unclear whether specific endothelial cells will also be required to ensure repopulation of the microvasculature, although some studies suggest that endothelial cells may be sufficiently plastic to become fated in vivo to this phenotype [84]. Likewise, grafting hepatoblasts with bipotentiality, i.e., differentiation not only into hepatocytes, but also biliary cells, may allow repopulation of the parenchyma, but also biliary tracks, with a single cell source. It is also encouraging to see that the combined seeding of hepatocytes/hepatoblasts and endothelium allows improved survival and function of the parenchymal cells [46,99,100]. 

Although we did not discuss this throughout the review, in the liver, there is not just one type of hepatocyte or LSEC; these are zonated, presenting a different phenotype when located in the periportal compared with the pericentral area of liver lobules. As this zonation is guided at least in part by the blood flow and delivery of oxygen, nutrients, and toxins [110], one might expect that this may occur at least in part spontaneously following grafting in vivo, even if this has not yet been demonstrated. Aside from blood flow, these specifications are also governed by local signals which may or may not be retained in the decellularized scaffold or provided by the neighboring grafted cells [111]. However, it will only become possible to address this question once we can create a more fully repopulated organ that survives for multiple weeks following grafting in vivo. 

### 7.3. Regulatory and Ethical Issues

Recellularized liver grafts which are produced according to GMP guidelines and shown to be effective and safe in animal experiments eventually proceed into clinical trials. Evaluation of safety and efficacy (i.e., biological benefit) of recellularized liver grafts would initially be tested in early-phase clinical trials. In early-phase trials, participants can be exposed to serious risks posed by transplanted grafts, such as adverse immunogenicity, infections and tumorigenesis. Therefore, defining a stage at which recellularized graft is launched into early-phase human trials is of utmost importance. Currently, there are no clinical and ethical guidelines to evaluate the safety of the recellularized liver grafts for early-phase clinical trials. Drug authorities such as the US Food and Drug Administration (FDA) and the European Medicines Agency (EMA) provide well defined and strict guidelines for pharmaceutical agents, but not cell-based bio-artificial organs such as decellularized liver grafts. Jongh et al. identified prominent themes to be considered for building clinical and ethical guidelines [112]. Defining cell source, risk–benefit assessment, criteria for patient selection, design of the trial, informed consent, oversight, and accountability issues related to cell-based bio-artificial organs (e.g., recellularized liver grafts) potentially benefit from clinical trials in adjacent fields such as cell therapy. 

## 8. Conclusions

The current state of perfusion decellularization technology allows for the manufacture of acellular liver scaffolds with an intact vascular lining and clinically relevant size. Concern regarding the potential immune reaction of the recipient body to decellularized matrices have been addressed, as indicated by the application of FDA-approved decellularized grafts in the clinic [22]. An intact vascular tree and/or biliary tree of the decellularized liver has been used to efficiently seed both parenchymal and non-parenchymal endothelial cells throughout the organ. The re-endothelialization of the vascular lumen of acellular liver scaffolds has been achieved using several techniques and using immortalized primary or stem-cell-derived endothelial cells to create grafts that allow in vivo perfusion. Repopulation of the parenchyma (both hepatocytes and biliary cells) is also possible using cell lines, primary adult or fetal cells, or stem-cell-derived progeny. Interestingly, repopulation of both compartments may enhance the survival and functionality of the parenchymal cells. Finally, reendothelialization of the vascular tree (with additional heparinization) enables recellularized livers to be grafted in both small and large animal models, at least in the short term. However, long-term maintenance of transplanted repopulated liver grafts has not yet been shown.

## Figures and Tables

**Table 1:** Rodent models in transplantation studies.

Reference	Infused Cells	Perfusion Culture	Vascular Patency Test	Implantation Duration
Takeishi et al. [90]	Human iPSC-derived hepatocyte- and cholangiocyte-like cells; primary human fetal and adult hepatocytes; primary rat hepatocytes and primary rat microvascular Ecs	36 h	NP	Up to 14 d
Bruinsma et al. [94]	Primary rat hepatocytes	5 d (24 h for transplantation studies)	Ex vivo perfusion with rat blood diluted in PBS (1:1), 2 h	24 h
Jiang et al. [69]	MSCs (bone marrow balb/c mice-derived)	4 weeks	NP	7 d
Pedro M. Baptista et al. [80]	MS1 endothelial Cells; HUVECs and human fetal liver cells (hFLCs)	3 d (MS1); 7 d (HUVECs and fHLCs)	Infusion fluorescein-labeled 250 kDa dextran particles; heparinized rat blood for 30 min	60 min
Ji Bao et al. [50]	Rat hepatocyte spheroids	Up to 5 d or 10 d for hepatocytes; or 5 d with hepatocyte-EC co-culture (24 h—post hepatocyte seeding)	Rat blood diluted with perfusion medium (hematocrit 20%) for 24 h	8 h

**Table 2:** Large animal models in transplantation studies.

Reference	Infused Cells	Perfusion Culture	Vascular Patency Test	Implantation Duration
Anderson et al. [100]	HUVECs and primary porcine hepatocytes	13–16 days	In vitro blood perfusion: freshly collected and heparinized porcine blood	48 h
Tahera Ansari et al. [70]	Porcine-adipose-derived MSCs	10 days	NP	70 and 90 min
Shaheen et al. [84]	HUVECs	Up to 30 d	In vitro blood perfusion: heparinized porcine blood; In vivo acute blood test by portal-venous blood flow of pig	Up to 20 d
In Kap Ko et al. [79]	MS1 endothelial cells	3 d	Heparinized porcine blood diluted in Kreb’s buffer, 3:1 ratio for 1 h.	1 d
Ji Bao et al. [95]	HUVECs and primary rat hepatocytes	NA	In vitro coagulation test by semiautomatic blood coagulation analyzer; Platelet adhesion and activation assay	1 h
Omar Barakat et al. [107].	Human fetal hepatocytes and fetal stellate cells	Up to 13 d	NP	2 h
